# Quantitative susceptibility mapping (QSM) and R2^*^ of silent cerebral infarcts in sickle cell anemia

**DOI:** 10.3389/fneur.2022.1000889

**Published:** 2022-10-20

**Authors:** Russell Murdoch, Hanne Stotesbury, Jamie M. Kawadler, Dawn E. Saunders, Fenella J. Kirkham, Karin Shmueli

**Affiliations:** ^1^Department of Medical Physics and Biomedical Engineering, University College London, London, United Kingdom; ^2^Developmental Neurosciences, UCL Great Ormond Street Institute of Child Health, London, United Kingdom; ^3^University Hospital Southampton NHS Foundation Trust, and Clinical and Experimental Sciences, University of Southampton, Southampton, United Kingdom

**Keywords:** quantitative susceptibility mapping (QSM), R2^*^, sickle cell anemia (SCA), composition, silent cerebral infarction (SCI), infarction, magnetic susceptibility

## Abstract

Silent cerebral infarction (SCI) is the most commonly reported radiological abnormality in patients with sickle cell anemia (SCA) and is associated with future clinical stroke risk. To date, there have been few histological and quantitative MRI studies of SCI and multiple radiological definitions exist. As a result, the tissue characteristics and composition of SCI remain elusive. The objective of this work was therefore to investigate the composition of segmented SCI lesions using quantitative MRI for R${}_{2}^{*}$ and quantitative magnetic susceptibility mapping (QSM). 211 SCI lesions were segmented from 32 participants with SCA and 6 controls. SCI were segmented according to two definitions (FLAIR+/–T1w-based threshold) using a semi-automated pipeline. Magnetic susceptibility (χ) and R${}_{2}^{*}$ maps were calculated from a multi-echo gradient echo sequence and mean SCI values were compared to an equivalent region of interest in normal appearing white matter (NAWM). SCI χ and R${}_{2}^{*}$ were investigated as a function of SCI definition, patient demographics, anatomical location, and cognition. Compared to NAWM, SCI were significantly less diamagnetic (χ = –0.0067 ppm vs. –0.0153 ppm, *p* < 0.001) and had significantly lower R${}_{2}^{*}$ (16.7 s^−1^ vs. 19.2 s^−1^, *p* < 0.001). SCI definition had a significant effect on the mean SCI χ and R${}_{2}^{*}$, with lesions becoming significantly less diamagnetic and having significantly lower R${}_{2}^{*}$ after the application of a more stringent T1w-based threshold. SCI-NAWM R${}_{2}^{*}$ decrease was significantly greater in patients with SCA compared with controls (–2.84 s^−1^ vs. –0.64 s^−1^, *p* < 0.0001). No significant association was observed between mean SCI–NAWM χ or R2^*^ differences and subject age, lesion anatomical location, or cognition. The increased χ and decreased R${}_{2}^{*}$ in SCI relative to NAWM observed in both patients and controls is indicative of lower myelin or increased water content within the segmented lesions. The significant SCI–NAWM R${}_{2}^{*}$ differences observed between SCI in patients with SCA and controls suggests there may be differences in tissue composition relative to NAWM in SCI in the two populations. Quantitative MRI techniques such as QSM and R${}_{2}^{*}$ mapping can be used to enhance our understanding of the pathophysiology and composition of SCI in patients with SCA as well as controls.

## Introduction

Covert or silent cerebral infarction (SCI) on magnetic resonance imaging (MRI) is the most commonly reported radiological abnormality in patients with sickle cell anemia (SCA) (1). SCI are lesions which appear hyperintense on T2-weighted Fluid Attenuated Inversion Recovery (FLAIR) MRI found in patients with normal neurological assessments (2). In SCA, the most common radiological definition of SCI was introduced in the Silent Infarct Transfusion (SIT) trial, where SCI were defined as a hyperintense lesion visible on at least two planes of a FLAIR image, measuring at least 3 mm in one dimension, and occurring in a subject without focal neurological symptoms (3). A more stringent SCI definition, applied in a study of adults with SCA, additionally required the lesion to appear hypointense on T1-weighted images (4).

Despite the absence of focal symptoms, the presence of SCI is associated with increased risk of future overt stroke (5) and, in some studies, with reduced full scale IQ (6–8). SCI may be secondary to cerebral small vessel disease (9), the mechanism considered likely in adults in the general population as they age (10). However, SCI mechanisms and pathophysiology remain poorly understood (11). The appearance of SCI on MRI is consistent with hypoxia-ischemia (insufficient oxygen and/or blood flow). Disease of the arterioles, microembolism, demyelination (12), and residual damage from venous sinus thrombosis (13) or posterior reversible encephalopathy syndrome have all been proposed as potential mechanisms. Risk factors include previous seizures, male sex, vascular stenosis, relatively low hemoglobin and high blood pressure (14). To date, few pathological studies of SCI have been reported and the tissue characteristics and composition of SCI remain elusive.

In most studies in patients with SCA, SCI has been studied as a binary variable. Few studies have considered measures such as lesion count or volume. Where these have been considered, lesion characteristics have been assumed to be homogenous across the brain, with all lesions having equivalent effects upon cognition and stroke risk. Our recent study attempted to further characterize lesions, beyond count and volume, by investigating the potential effects of additional factors such as lesion depth in white matter and lesion brain lobe on the relationship between SCI and cognition (15). We found no significant relationship between any global measures of SCI burden and cognitive impairment in patients with SCA, irrespective of SCI definition. This study agrees with other recent studies, which suggest that additional factors such as MRI field strength and FLAIR image resolution may affect the relationship between SCI and cognition, along with patient characteristics and treatment regimens (16, 17).

The objective of this study was to build on that initial research, using transverse relaxation rate (R${}_{2}^{*}$) and quantitative susceptibility mapping (QSM) to investigate SCI composition and lesion heterogeneity. We investigated the magnetic susceptibility (χ) and R${}_{2}^{*}$ of SCI relative to normal appearing white matter (NAWM) in the contralateral hemisphere. QSM calculates the spatial distribution of χ from the phase images acquired using gradient echo MRI (18). χ is an intrinsic property of tissue, determined by tissue composition and microstructure (19). In white matter, where most SCI occur (11), myelin is the key susceptibility source and is relatively diamagnetic. However, iron (paramagnetic, primarily in ferritin), calcifications (diamagnetic) and deoxygenated hemoglobin (paramagnetic) may also contribute to the measured χ in white matter regions (19). We chose to incorporate R${}_{2}^{*}$ into this study as it can be calculated from the gradient echo magnitude data acquired for QSM and provides complementary information to QSM. That is, R${}_{2}^{*}$ increases as the total number of microscopic susceptibility sources in a voxel increases, irrespective of whether the sources are paramagnetic or diamagnetic. In other words, tissue R2^*^ would decrease if there was a loss of diamagnetic molecules whilst χ would increase. This means that R${}_{2}^{*}$ can be used together with QSM to disambiguate changes in positive and negative χ sources (20, 21).

More specifically, the two main aims of this study were to: 1) examine the χ and R${}_{2}^{*}$ of SCI lesions relative to NAWM; and 2) investigate lesion χ and R${}_{2}^{*}$ as a function of other lesion characteristics, participant demographics and cognition.

## Materials and methods

### Patients

Patients with SCA (hemoglobin-SS) and age and race-matched healthy control (HC) participants were recruited to two studies with overlapping MRI protocols between 2016 and 2019: the Sleep Asthma Cohort follow-up (SAC) (22) and the Prevention of Morbidity in Sickle Cell Anemia baseline investigation (POMS) (23). Exclusion criteria have been reported previously (24).

Ethical approval was granted by West London NHS (05/Q0408/42, 11/EM/0084, 15/LO/0347), Yorkshire NHS (15/YH/0213) and University College London (14475/001) ethics committees. Fully informed consent and assent according to the Declaration of Helsinki were obtained from participants and, for children, from their parent/guardian.

### MRI acquisition

All patients were imaged on a 3T Siemens (Erlangen, Germany) Magnetom Prisma MRI system using a 64-channel head coil. The MRI protocol included: a T2-weighted fluid attenuated inversion recovery (FLAIR) sequence for SCI identification and definition [voxel dimensions = 0.65 × 0.65 × 1 mm^3^, matrix size = 306 × 384 × 240, repetition time (TR) = 5,000 ms, echo time (TE) = 395 ms, inversion time (TI) = 1,800 ms], a T1-weighted magnetization-prepared rapid gradient-echo (MP-RAGE) sequence for white matter segmentation and SCI definition [voxel dimensions = 1 × 1 × 1 mm^3^, 256 × 240 × 256, TE_1_ = 2.74 ms, TR = 2,300 ms, flip angle = 8°], and a multi-echo gradient echo (ME-GRE) sequence used to calculate R${}_{2}^{*}$ and χ maps [voxel dimensions=1.15 × 1.15 × 1.15 mm^3^, matrix size = 180 × 220 × 166, echoes = 7, TE_1_ = 3 ms, ΔTE = 4 ms, flip angle = 15°].

### SCI segmentation

The full details of the SCI segmentation pipeline have been published previously (15) and are described briefly below. Each FLAIR image was assessed by an experienced neuroradiologist (D.S.) who identified SCI and recorded the lobe in which SCI were located. The SIT trial definition was used; an area of hyperintense signal, measuring at least 3 mm in greatest dimension, and occurring in a patient with no focal neurological symptoms. A generous region of interest (ROI) was manually drawn over the region of increased signal. As in prior studies, a minimum intensity threshold, derived from the mean cortical FLAIR intensity (1.02·Mean_FLAIR − cortex_) (25), was applied to the ROI to remove voxels which did not appear hyperintense on the FLAIR image.

**Figure 1:**
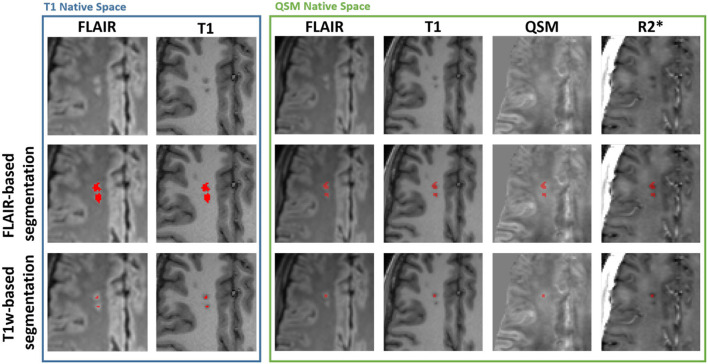
Example silent cerebral infarct segmentations. Blue Box: Axial view of two silent cerebral infarcts (SCI) segmented in a representative patient with sickle cell anemia (SCA) overlaid on FLAIR and T1-weighted images in the native T1w space. The example SCI appear hyperintense on FLAIR images and hypointense on T1-weighted images. The application of a more stringent T1w-based maximum intensity threshold (bottom) reduces the volume of the segmented SCI lesions relative to the standard FLAIR-based segmentation (middle). Green Box: Axial view of the same SCI after interpolation into the native quantitative susceptibility mapping (QSM) space, overlaid over FLAIR, T1w, QSM and R2* images. The SCI appear bright on QSM and dark on R2* maps relative to normal appearing white matter. Note that the coregistration of the lesions into the QSM space results in the lower SCI segmentation appearing in the adjacent slice.

To meet the more stringent SCI definition (4, 26), a second maximum intensity threshold, derived from the mean cortical T1-weighted MP-RAGE intensity (1.02·Mean_T1 − cortex_) was applied to the ROI to remove voxels which did not appear hypointense on the T1 image. Example SCI segmentations based upon the two definitions are shown in Figure 1.

NAWM ROIs in the contralateral hemisphere were segmented by mirroring each segmented SCI across the brain midline and shifting the segmentation to a manually selected position in NAWM in the same transverse slice, avoiding any SCI in the contralateral hemisphere.

### Exclusion criteria

Lesions which consisted of 2 voxels or fewer after interpolation into the native QSM space were excluded from this study as they were no longer considered to meet the requirement of measuring 3 mm in at least one dimension.

### MRI image processing

QSM was calculated from the ME-GRE images using the following pipeline: Coil combination was performed using a phase difference approach (27). Total field maps were estimated from a non-linear fit of the reconstructed complex images (28), with residual wraps in the fitted field removed using SEGUE phase unwrapping (29). Images were realigned with the main magnetic field direction (30) prior to background field removal carried out using projection onto dipole fields (31). The χ maps were calculated using the iterative Tikhonov method (32), with regularization parameter (α = 0.013) selected using L-curve methods in six representative subjects (33). R${}_{2}^{*}$ maps were calculated from a linear fit of the log-transformed ME-GRE magnitude images as a function of echo time. QSM and R${}_{2}^{*}$ image processing was performed using MATLAB (MATLAB 2021b, MathWorks Inc., Natick, MA, USA).

Cortex, ventricle and white matter segmentations were obtained using Freesurfer, applied to the T1-weighted MP-RAGE images (34, 35). The white matter segmentation was further subdivided into juxtacortical, periventricular, or deep voxels by calculating the respective distance between the voxels and the cortex and ventricle Freesurfer segmentations. Juxtacortical voxels were defined as the 75th percentile in the distance to the cortex map and periventricular voxels were defined as the 5th percentile in the distance to the ventricle map (15). The separate white matter segmentations were used to classify the depth of SCI within white matter.

FLAIR images were affinely registered to their corresponding T1-weighted MP-RAGE using FSL FLIRT (36, 37). The T1-weighted MP-RAGE images were affinely registered to the tilt-corrected first echo ME-GRE magnitude image and the resultant affine transformation was applied to each of the SCI/contralateral NAWM segmentations (before and after the application of the additional T1w threshold), and the juxtacortical/periventricular/deep white matter maps with nearest neighbor interpolation.

### Cognitive assessment

Participants enrolled in the SAC study who were over 16 years old underwent the Wechsler Adult Intelligence Scale (WAIS) test to assess their cognitive performance. The WAIS test is designed to measure full scale IQ (FSIQ) using a series of tests across several domains (38). WAIS also provides measures of working memory index (WMI), reflecting the ability to retain and manipulate information over a short period, and processing speed index (PSI), reflecting mental speed. Participants younger than 16 years completed the equivalent Wechsler Intelligence scale for Children (WISC) (39). Participants enrolled in the POMS study completed the Wechsler Abbreviated Scale of Intelligence (WASI) (40), a shorter form of the WAIS/WISC which provides comparable results (41), as well as the WAIS/WISC subtests necessary to measure PSI and WMI.

### Statistical analysis

All statistical analyses were performed in R Statistical Software. (v4.1.2; R Core Team 2021). Group-wise differences in Age/FSIQ/WMI/PSI between patients with SCA and controls were examined using Wilcoxon Rank Sum Tests, and sex differences were considered using Chi-Squared tests.

ROI mean χ and R${}_{2}^{*}$, and voxel count were calculated within each lesion before and after the application of the T1w maximum intensity threshold in the native QSM space. For each SCI/NAWM ROI the mean χ and R${}_{2}^{*}$ and voxel count were calculated within each of the three white matter segmentations, i.e., for SCI with voxels in multiple WM regions (e.g., deep and juxta-cortical) separate mean values were calculated for the two WM regions.

Mean SCI χ and R${}_{2}^{*}$ were investigated relative to the mean values measured within NAWM in the contralateral hemisphere. Mean values in the SCI and NAWM were compared using correlation, Bland-Altman analysis, and paired *t*-tests. Paired *t*-tests were applied to consider differences in the mean SCI χ and R${}_{2}^{*}$ before and after the application of the T1w-based intensity threshold.

Analysis of variance (ANOVA) was used to investigate whether subject age (log-transformed), sickle cell status, brain lobe and lesion volume (log-transformed) had a significant effect on the measured SCI χ and R${}_{2}^{*}$.

Groupwise differences between age-corrected SCI χ and R${}_{2}^{*}$ in patients with SCA and controls were examined using student's *t*-test. *Post-hoc* differences in age-corrected SCI χ and R${}_{2}^{*}$ as a function of brain lobe were investigated using ANOVA and Tukey's honest significance test. Correlation analysis was used to investigate the association between lesion volume and age-corrected SCI χ and R${}_{2}^{*}$, The effect of white matter depth on age-corrected SCI χ and R${}_{2}^{*}$ was considered in a separate ANOVA, as some lesions were split across multiple white matter regions. In the SCA cohort only, for whom blood samples were available, correlations were examined between age-corrected SCI χ and R${}_{2}^{*}$ and blood hemoglobin levels, a measure of anemia.

To consider the potential association between cognitive performance and mean SCI χ/R${}_{2}^{*}$, mean χ/R${}_{2}^{*}$ were calculated across SCI in participants with multiple SCI. Correlations were then investigated between mean SCI χ/R${}_{2}^{*}$ per subject and measures of cognitive performance.

## Results

A total of 93 participants with SCA and 33 controls were eligible for inclusion in this investigation after being imaged with the three necessary MRI sequences (T1w MP-RAGE, T2w FLAIR, and ME-GRE) as part of the two clinical studies (Table 1). The presence of SCI was identified in 38/93 SCA subjects (40.8%) and 10/33 controls (3 Hb-AA, 7 Hb-AS) (30.3%). 2 patients with SCA and 1 control were excluded due to poor image quality in at least one of the three image contrasts. SCI in 4 patients with SCA and 3 controls did not meet the inclusion criteria after interpolation into the native QSM space. As a result, SCI segmented in 32 patients with SCA, and 6 controls were considered in this study.

**Table 1:** Overview of participant and lesion characteristics in patients with sickle cell anemia (SCA) and healthy controls (HC).

**Participants**	**SCA**	**HC**	**P**
N	32	6	–
Male (Yes/No)	22/10	2/4	0.1
Age (mean ± sd) [years]	20.6 ± 13.1	24.5 ± 17.4	0.54
Age (min-max) [years]	8.5-63.9	14.8-59.5	–
Hematocrit (mean ± sd) (g/dL)	8.82 ± 1.47	NA	–
Chronic transfusion (yes|no)	2|30	NA	–
Transfusion last 6 Months	4|28	NA	–
Abnormal MRA (yes|no)	7|25	0|6	–
Severe vasculopathy (yes|no)	1|31	0|6	–
FSIQ (mean ± sd)	91.9 ± 13.5	89.5 ± 7.4	0.52
WMI (mean ± sd)	89.6 ± 13.0	94.7 ± 7.8	0.48
PSI (mean ± sd)	87.6 ± 11.1	86 ± 19.0	0.55
Lesion count (mean ± sd)	5.6 ± 5.8	5.5 ± 4.1	0.75
Lesions (FLAIR|T1w)	
N	178|44	33|3	–
Frontal	113|35 (63.5|79.5%)	12|1 (36.4|33.3%)	–
Occipital	3|0 (1.7|0.0%)	5|1 (15.2|33.3%)	–
Parietal	53|6 (29.8|13.6%)	13|1 (39.4|33.3%)	–
Temporal	4|0 (2.2|0.0%)	3|0 (9.1|0.0%)	–
Other	4|3 (2.2|4.5%)	0|0 (0.0|0.0%)	–
Right Hemisphere	92|25 (51.7|56.8%)	9|1 (27.3|33.3%)	–
Left Hemisphere	86|19 (48.3| 43.2%)	24|2 (72.7|66.7%)	–

Lesions characteristics are shown for both the FLAIR-based and T1-weighted-based SCI segmentations. *P*-values are reported from the Wilcoxon Rank Sum Tests and Chi-Squared tests used to compare differences in the SCA and HC cohorts.

211 SCI Segmentations Persisted after Interpolation into the native QSM space, 178 (~84%) in patients with SCA and 33 (~16%) in controls. Most lesions were in either the frontal (125/211, 59.2%) or parietal lobe (66/211, 31.3%), with fewer SCI located in the occipital (8/211, 3.8%) or temporal lobe (7/211, 3.3%). Heatmaps showing the distribution and frequency of SCI segmented in the SCA and control subjects in this cohort has been published previously (15). Lesion distributions were found to be similar in the patient and control groups.

Of the 211 lesion segmentations which persisted after interpolation into the native QSM space, 47 (~22%) persisted after application of the additional, more stringent T1w-based threshold. Of these, 44/47 (~94%) were present in sickle cell subjects and 3/47 (~6%) were present in healthy controls. Following application of the T1w-based threshold, a much lower proportion of lesions persisted in the parietal lobe (7/66, 10.6%) compared with the frontal lobe (36/125, 28.8%).

**Figure 2:**
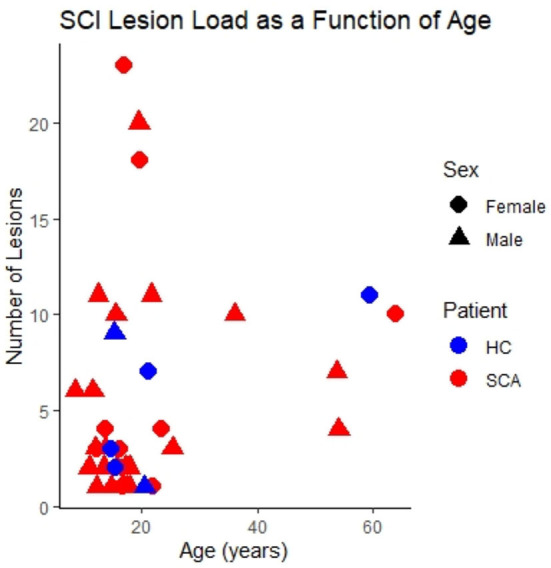
SCI lesion count. The number of silent cerebral infarcts (SCI) segmented in each participant as a function of age, sex, and sickle cell status in the study cohort. Lesions were defined according to the FLAIR-based SIT trial definition. This is the case for all subsequent figures unless indicated otherwise.

The distribution of SCI as a function of age, sex and SCA status within the study cohort is shown in Figure 2.

### Comparison of χ and R${}_{2}^{*}$ in SCI vs. NAWM

**Figure 3:**
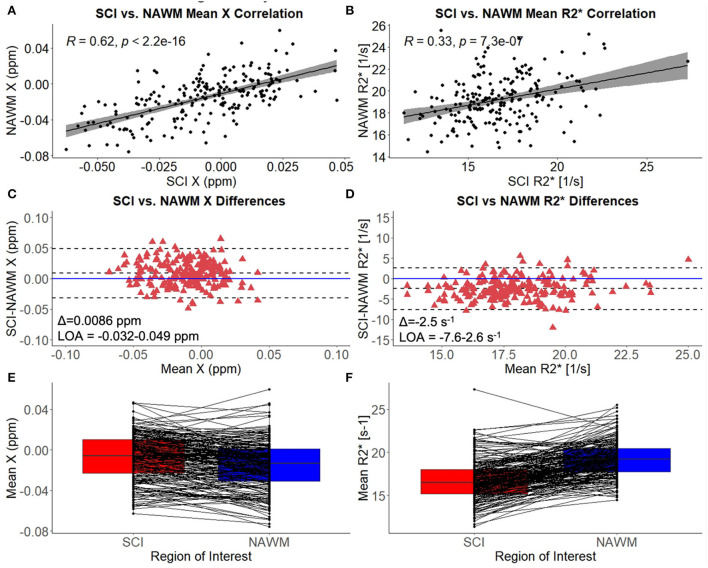
SCI vs. NAWM χ and R${}_{2}^{*}$. **(A)** Correlation between the mean susceptibility (χ) in segmented silent cerebral infarcts (SCI) and contralateral normal appearing white matter (NAWM). **(B)** Correlation between mean SCI and NAWM R${}_{2}^{*}$. **(C)** Bland-Altman analysis of SCI-NAWM χ differences. The mean bias (Δ) is positive suggesting that on average the SCI are less diamagnetic than NAWM. **(D)** Bland-Altman analysis of SCI-NAWM R${}_{2}^{*}$ differences. The mean bias is negative indicating that on average the SCI have lower R${}_{2}^{*}$ than NAWM. Bland-Altman plots also show the limits of agreement (LOA) and the horizontal blue line is located at SCI-NAWM = 0. **(E)** Boxplot comparing mean SCI and NAWM χ, showing that mean χ is significantly elevated in SCI. **(F)** Boxplot showing that mean R2* is significantly reduced in SCI relative to NAWM.

Mean χ (and R${}_{2}^{*}$) values were strongly correlated between SCI and corresponding NAWM ROIs (χ: *r* = 0.62, *p* < 0.001, R${}_{2}^{*}$: *r* = 0.33, *p* = < 0.001) (Figures 3A,B). Paired t-tests indicated that mean χ was significantly higher (less diamagnetic) in SCI compared to NAWM in the contralateral hemisphere (SCI: – 0.0067 ppm vs. NAWM: – 0.0153 ppm, Δ = 0.0086 ± 0.0014 ppm, *p* < 0.001), while mean R${}_{2}^{*}$ was significantly reduced (SCI: 16.7 s^−1^ vs. 19.2 s^−1^, Δ = – 2.5 ± 0.2 s^−1^, *p* < 0.001). The mean and distribution of differences between SCI and NAWM are shown in the Bland-Altman plots in Figures 3C,D and the boxplots in Figures 3E,F.

### Investigating SCI χ and R${}_{2}^{*}$ as a function of lesion definition

Figure 1 demonstrates that the signal abnormalities in the FLAIR images extend beyond regions of hypointense signal in the T1–weighted images. Applying the T1w-based threshold reduced the mean SCI volume size by ~86% (FLAIR threshold: 118.7 ± 217.5 mm^3^ vs. FLAIR + T1-w threshold: 16.7 ± 43.2 mm^3^).

Prior to T1w-based thresholding, no significant difference in SCI–NAWM χ difference was found between lesions which did and did not persist using the stricter definition. (Δχ _Persistent_: 0.0095 ppm vs. Δχ _Non − persistent_: 0.0084 ppm, *p* = 0.7). A trend toward lower SCI–NAWM R${}_{2}^{*}$ differences was observed in lesions which did persist using the more stringent definition relative to those that did not (Δ R${}_{2}^{*}$_Persistent_: –3.14 s^−1^ vs. Δ R${}_{2}^{*}$_Non−*persistent*_: –2.31 s^−1^, *p* = 0.06). Bland Altman analysis comparing SCI and NAWM χ and R${}_{2}^{*}$ in the lesions which did and did not persist after the application of the T1w-based threshold is shown in Supplementary Figure 1.

**Figure 4:**
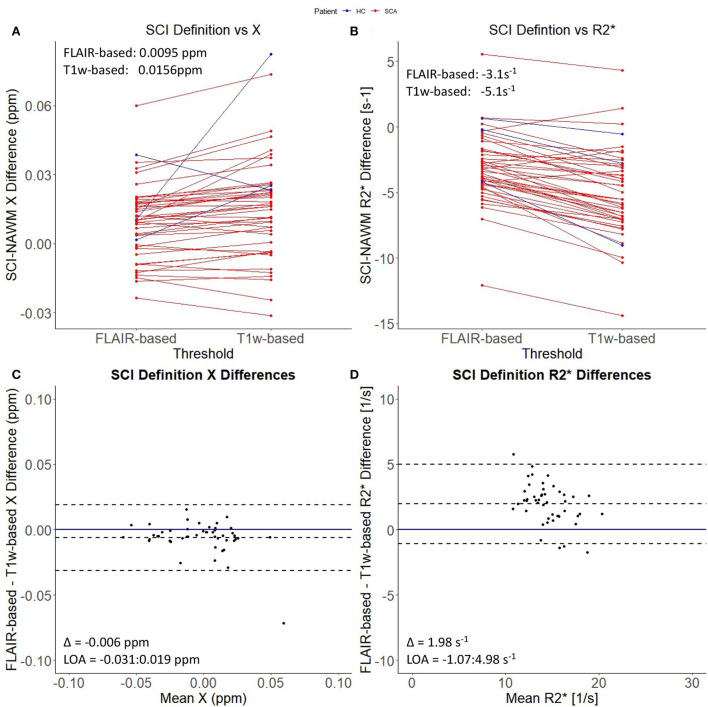
Effect of SCI definition on SCI-NAWM χ and R${}_{2}^{*}$. **(A)** Comparison of mean silent cerebral infarct (SCI) and normal appearing white matter (NAWM) susceptibility (χ) differences before and after the application of the more stringent T1w-based threshold. **(B)** Comparison of mean SCI-NAWM R2* differences before and after application of the T1w-based threshold. Mean SCI-NAWM χ and R${}_{2}^{*}$ differences for the two definitions are shows in **(A,B)** respectively. **(C)** Bland-Altman analysis of mean SCI-NAWM χ differences before and after application of the T1w-based threshold. The mean bias (Δ) and limits of agreement (LOA) are annotated within the plot. **(D)** Bland-Altman analysis of the mean SCI-NAWM R${}_{2}^{*}$ differences.

In the lesions which did persist, paired *t*-tests showed that, after thresholding, lesion χ was significantly less diamagnetic in the T1w thresholded ROIs relative to the FLAIR segmentations (SCI χ _T1w_: 0.001 ppm vs. SCI χ _FLAIR_: – 0.005 ppm, *p* = 0.002) which manifests as larger χ differences relative to NAWM (Δ χ _T1w_: 0.0156 ppm vs Δ χ _FLAIR_: 0.0095 ppm, Δ = 0.0061 ± 0.0019 ppm, *p* = 0.002) (Figure 4). Similarly, SCIs defined using the T1w-based threshold had significantly lower mean R${}_{2}^{*}$ (SCI R${}_{2\text{T}1\text{w}}^{*}$: 13.6 s^−1^ vs. SCI R${}_{2\text{FLAIR}}^{*}$: 15.6 s^−1^, Δ = 2.0 ± 0.2 s^−1^, *p* < 0.001), corresponding to larger SCI–NAWM R${}_{2}^{*}$ differences (Δ R${}_{2\text{T}1\text{w}}^{*}$: −5.1 s^−1^ vs Δ R${}_{2\text{FLAIR}}^{*}$: −3.1 s^−1^).

### Investigating SCI χ and R${}_{2}^{*}$ as a function of subject demographic, anatomical location and volume

The results of the ANOVA investigating the effect of subject age, sickle cell status, brain lobe and lesion volume upon SCI χ and R${}_{2}^{*}$ are shown in Table 2.

**Table 2:** Results of an ANOVA investigating the effect of subject age, sickle cell status, brain lobe and lesion volume on SCI and NAWM χ and R${}_{2}^{*}$.

**ANOVA**	**LN (Age)**	**SCA**	**Lobe**	**Ln (Volume)**
SCI χ	**0.0129**	0.0756	**< 0.0001**	0.0815
NAWM χ	**0.0045**	0.15	**0.0002**	0.87
SCI-NAWM χ	0.48	0.85	**0.037**	0.11
SCI R_2_*	**0.009**	**< 0.0001**	0.16	**0.01**
NAWM R_2_*	**0.007**	0.26	0.07	0.08
SCI-NAWM R_2_*	0.997	**< 0.001**	0.09	0.45

Presented in the table are *p*-values indicating which variables were significantly associated with differences in SCI and NAWM χ and R${}_{2}^{*}$ values.

*P* < 0.05 were considered significant and are highlighted in bold.

**Figure 5:**
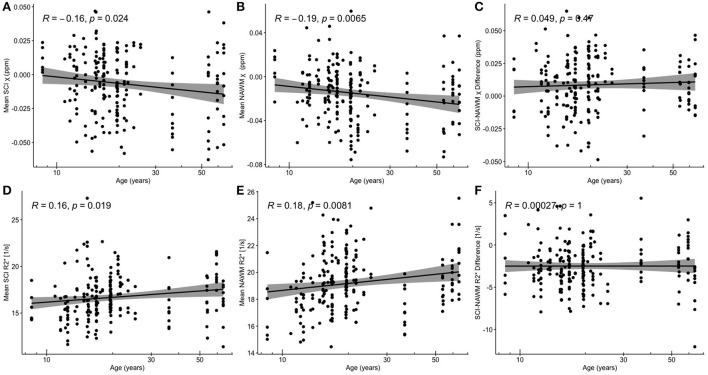
Correlations between SCI and NAWM χ and R${}_{2}^{*}$ and age. **(A)** Mean silent cerebral infarct (SCI) susceptibility (χ) **(B)** mean normal appearing white matter (NAWM) χ **(C)** SCI-NAWM χ difference **(D)** mean SCI R${}_{2}^{*}$(E) mean NAWM R2*(F) mean SCI-NAWM R2* difference as a function of age plotted on a logarithmic scale. SCI were segmented using the FLAIR-based SIT trial definition.

Figure 5 shows SCI and NAWM χ and R${}_{2}^{*}$ plotted as a function of log-transformed age. SCI and NAWM χ were both significantly negatively correlated with log-transformed subject age, consistent with increasing myelin content in this relatively young cohort (42). However, no significant association with age was observed for the SCI–NAWM χ difference (Figure 5). In contrast, SCI and NAWM mean R${}_{2}^{*}$ were positively associated with age, in agreement with increased myelin content, and no significant correlation was observed in the SCI–NAWM R${}_{2}^{*}$ difference.

When considering the relationship between SCI–NAWM χ and R${}_{2}^{*}$ in SCI which persisted on application of the T1w threshold, no significant correlation between χ / R${}_{2}^{*}$ and age was observed (Supplementary Figure 2). In subsequent results the SCI and NAWM χ and R${}_{2}^{*}$ are age-corrected to remove any confounding effects of age on the effect of SCA, brain lobe and lesion volume on χ and R${}_{2}^{*}$ values.

**Figure 6:**
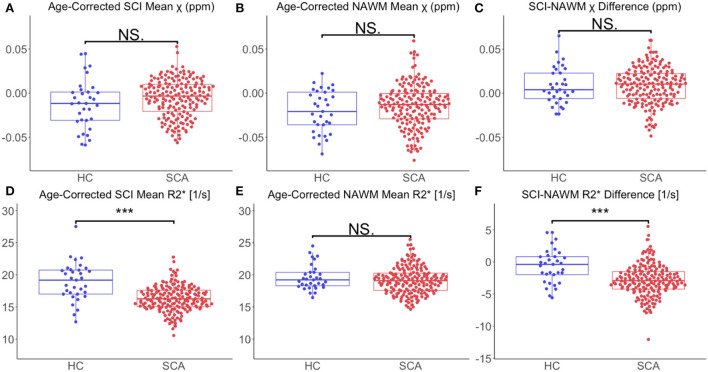
Effect of SCA on SCI and NAWM χ and R${}_{2}^{*}$. **(A)** Comparison of mean age-corrected silent cerebral infarct (SCI) susceptibility (χ) in patients with sickle cell anemia (SCA) and healthy controls (HC). **(B)** Comparison of mean age-corrected normal appearing white matter (NAWM) χ in SCA and HC. **(C)** No significant differences in mean SCI-NAWM χ difference were observed between the SCA and HC groups (0.009 vs. 0.006 ppm, *p* =0.5). **(D)** Comparison of mean age-corrected SCI R${}_{2}^{*}$ in SCA and HC. **(E)** Comparison of mean age-corrected NAWM R${}_{2}^{*}$ in SCA and HC. **(F)** The SCI–NAWM R${}_{2}^{*}$ difference was significantly lower in patients with SCA relative to HC (– 2.84 vs. – 0.64 s^−1^, *p* < 0.0001). SCI were segmented using the FLAIR-based SIT trial definition. ***p < 0.001.

Comparing age-corrected SCI and NAWM χ in patients with SCA relative to controls, no significant difference was observed in the SCI mean χ (SCI χ _SCA_: – 0.006 ppm vs. SCI χ _HC_: – 0.012 ppm, *p* = 0.11), or the SCI–NAWM differences (Δ χ _SCA_: 0.0087 ppm vs. Δ χ _HC_: 0.0085 ppm, *p* = 0.96). Age-corrected SCI R${}_{2}^{*}$ was significantly lower in patients with SCA relative to controls (SCI R${}_{2\text{SCA}}^{*}$: 16.3 s^−1^ vs. SCI R${}_{2\text{HC}}^{*}$: 18.9 s^−1^, *p* < 0.0001), and this was also reflected in the SCI–NAWM difference (Δ R${}_{2\text{SCA}}^{*}$: −2.84 s^−1^ vs Δ R${}_{2\text{HC}}^{*}$: −0.64s^−1^, *p* < 0.0001) (Figure 6). Participant numbers precluded further comparisons of the T1w-thresholded lesions between patients with SCA and controls, with such lesions only persisting in 2 controls.

**Figure 7:**
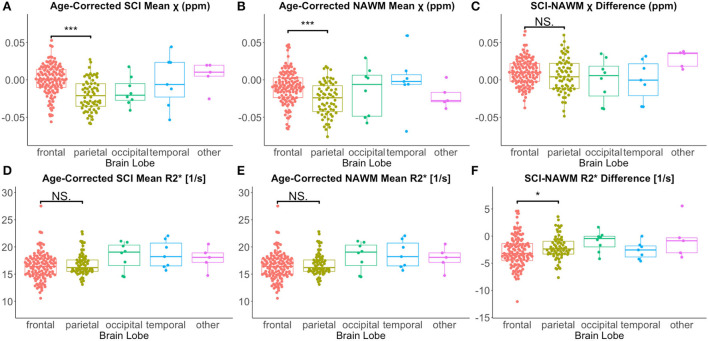
Effect of anatomical location on SCI and NAWM χ and R${}_{2}^{*}$. Comparison of mean susceptibility (χ) and R${}_{2}^{*}$ measured in silent cerebral infarcts (SCI) in each of the brain lobes. **(A)** SCI in the frontal lobe were significantly more paramagnetic relative to lesions in the parietal lobe. **(B)** This lobar χ difference was also observed in normal appearing white matter (NAWM). **(C)** This resulted in no significant differences in the SCI–NAWM susceptibility difference between the lobes. **(D,E)** No significant differences were observed between lobes in the SCI and NAWM mean R2* comparisons. **(F)** A small, significant difference was observed between the SCI–NAWM R${}_{2}^{*}$ difference between lesions in the frontal and parietal lobes. SCI were segmented using the FLAIR-based SIT trial definition. *p < 0.05. ***p < 0.001.

Lesion χ and R${}_{2}^{*}$ were investigated as a function of brain lobe. Increased χ within SCI in the frontal lobe, relative to the parietal lobe, mirrored increased χ observed in NAWM, and no significant SCI–NAWM χ differences were observed between brain lobes (Figure 7). Inter-lobe mean R${}_{2}^{*}$differences were not observed, but SCI–NAWM R${}_{2}^{*}$ differences were slightly elevated in the frontal vs parietal lobe (Δ R${}_{2\text{frontal}}^{*}$: – 2.86 s^−1^ vs. Δ R${}_{2\text{parietal}}^{*}$: – 2.13 s^−1^, *p* = 0.05).

Similar χ trends as for the FLAIR defined lesions were observed between T1w-thresholded lesions in the frontal and parietal lobes (Supplementary Figure 3). However, the between-lobe SCI–NAWM Δ R${}_{2}^{*}$ difference between the frontal and parietal lobes observed for FLAIR lesion segmentations was no longer statistically significant for the more stringently defined T1w-thresholded SCI.

**Figure 8:**
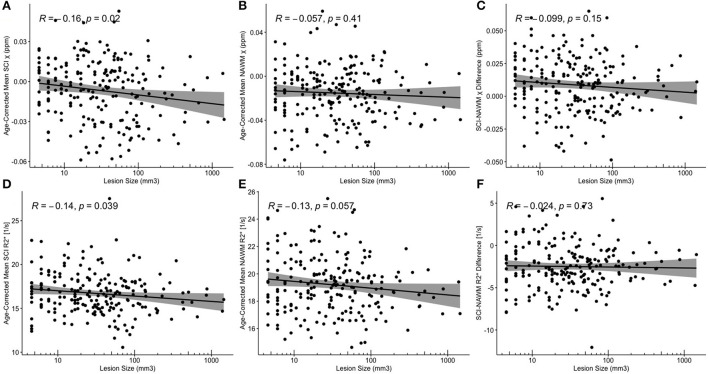
Correlations between SCI and NAWM χ and R${}_{2}^{*}$ and lesion volume. **(A,D)** Mean silent cerebral infarct (SCI) **(B,E)** normal appearing white matter (NAWM) **(C,F)** SCI-NAWM differences in susceptibility (χ) and R2* as a function of lesion volume plotted on a logarithmic scale. Mean SCI χ and R2* in regions segmented based upon the FLAIR-based definition.

Despite significant correlations between age-corrected SCI χ and R${}_{2}^{*}$ and log (lesion volume) in the FLAIR-defined SCI, no significant associations between SCI–NAWM differences in χ (and R${}_{2}^{*}$) and lesion size were found (Figure 8). Furthermore, no significant relationship between the age-corrected SCI–NAWM χ and R${}_{2}^{*}$ difference and white matter depth were observed for the juxta-cortical, deep, or periventricular SCI (Supplementary Figure 5).

No significant associations were found between age-corrected SCI mean χ and R${}_{2}^{*}$ (or SCI-NAWM χ and R${}_{2}^{*}$ differences) and blood hemoglobin, a measure of anemia severity only available in patients with SCA.

### Investigating effect on cognitive impairment as a function of SCI χ and R${}_{2}^{*}$

**Figure 9:**
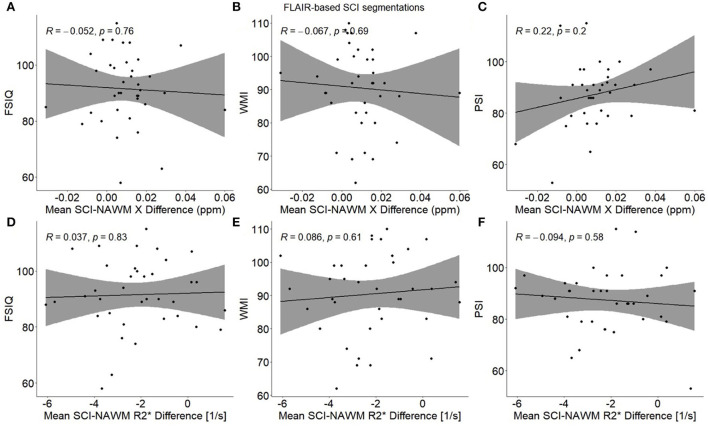
Association between participant mean SCI–NAWM χ and R${}_{2}^{*}$ differences and measures of cognitive performance. Within each participant, mean silent cerebral infarcts (SCI) χ and R${}_{2}^{*}$ differences relative to normal appearing white matter (NAWM) were calculated across all lesions, and associations were examined with full scale IQ (FSIQ) **(A,D)**, working memory index (WMI) **(B,E)** and processing speed index (PSI) **(C,F)**. No significant associations were observed between SCI–NAWM χ and R${}_{2}^{*}$ and any measures of cognition. SCI were segmented using the FLAIR-based SIT trial definition.

No significant associations were found between any of the cognitive measures (FSIQ/WMI/PSI) and mean SCI–NAWM χ or R${}_{2}^{*}$ differences (or mean age-corrected SCI χ and R${}_{2}^{*}$) when considering the mean lesion values in participants with multiple lesions (Figure 9). No associations were found for either the combined SCA and HC cohort, or in the SCA cohort only.

## Discussion

Using QSM and R${}_{2}^{*}$, the objectives of this study were to investigate the composition of SCI relative to NAWM in patients with SCA and controls and investigate SCI χ and R${}_{2}^{*}$ as a function of other lesion characteristics, participant demographics and cognition. Mean χ was significantly higher (less diamagnetic) and mean R${}_{2}^{*}$ was significantly reduced in SCI compared to NAWM in the contralateral hemisphere. Lesion χ was significantly less diamagnetic with significantly lower mean R${}_{2}^{*}$in the persisting T1w- thresholded ROIs relative to the FLAIR-based segmentations. SCI-NAWM R${}_{2}^{*}$ differences were larger in patients with SCA relative to controls. There was no effect of lesion size or anatomical location and no association with cognitive performance.

The rate of SCI within patients with SCA eligible for inclusion in this study (40.8%) was in line with rates reported previously in SCA literature (43). SCI were reported in 30.3% of healthy control subjects, which may seem higher than expected given the age range of controls in this cohort. However, previous high resolution MRI studies of SCI, with similar age ranges of controls, have reported high prevalence of SCI within the control cohorts, as high as 70% (44).

### Comparison of χ and R${}_{2}^{*}$ in SCI vs NAWM

QSM and R${}_{2}^{*}$ mapping showed increased mean χ and reduced mean R${}_{2}^{*}$ within SCI segmented using the SIT-trial (FLAIR-based) definition relative to NAWM in the contralateral hemisphere. This can be seen visually in Figure 1, with the segmented SCI appearing brighter on χ maps and darker on R${}_{2}^{*}$ maps relative to the surrounding NAWM.

An increased mean χ relative to NAWM could be caused by either an increase in the concentration of paramagnetic substances, such as iron in the form of ferritin or deoxygenated hemoglobin, or a reduction in diamagnetic χ sources such as myelin. The reduction of R${}_{2}^{*}$ in SCI suggests a loss of susceptibility sources (less myelin) as opposed to additional χ sources, since increased iron accumulation would be expected to increase R${}_{2}^{*}$ within the lesions.

Cerebral infarction is the process of tissue death caused by ischemia and hypoxia which results in a region of necrotic tissue. These necrotic regions are believed to be what is segmented based upon the accepted definitions in the SCA literature. As necrosis results in cell swelling, membrane rupture and the release of cell contents into the extracellular space triggering an inflammatory response, it is reasonable that susceptibility sources are reduced within the SCI, with loss of myelin, either primary or secondary to infarction, a potential explanation for our observations.

Ischemia and hypoxia have been linked to brain iron accumulation. Ischemia may be a key inflammatory trigger in SCA, and inflammatory processes have been linked to iron accumulation in numerous neurodegenerative conditions including Parkinson's (45–47) and Alzheimer's (48, 49). However, it appears that iron is preferentially taken up in the deep gray matter regions, where iron is predominately stored within ferritin macromolecules, as opposed to regions surrounding the sites of infarction (50).

Changes in myelin content may not be the sole contributor to the χ and R${}_{2}^{*}$ changes observed in SCI. Increased water content causes similar χ and R${}_{2}^{*}$ changes relative to normal appearing white matter, as reduced myelin content. Increased tissue water content causes the bulk tissue χ to become closer to zero in the calculated χ maps (which are intrinsically referenced to the χ of water). Therefore, in white matter, increased tissue water content causes the bulk χ of the regions to increase (become less diamagnetic). The lower concentration of susceptibility sources, caused by excess water, reduces R${}_{2}^{*}$ relative to NAWM, comparable to loss of myelin content. Regions of increased water content, in conditions such as edema, appear bright on T2-weighted MRI sequences and dark on T1-weighted MRI sequences relative to NAWM, analogous to regions of infarction. Therefore, it is challenging to separate the two mechanisms. Future imaging studies should aim to differentiate between increased water content and regions of infarction using diffusion weighted imaging, where edema appears bright, or CT imaging where edema results in reduced attenuation relative to normal appearing white matter.

### Investigating SCI χ and R${}_{2}^{*}$ as a function of lesion definition

The most common (FLAIR-based) definition of SCI, developed for the SIT trial, required a signal abnormality measuring at least 3 mm in greatest dimension and visible in at least two planes (3). This definition is dependent upon acquisition parameters such as voxel dimensions, which will determine whether lesions are visible in two planes or not, depending on the size of the lesion relative to the voxel dimensions. Therefore, advances in MRI hardware and acquisition have resulted in increased sensitivity to signal abnormalities compared with images obtained when the definitions were published.

In this study, all hyperintense signal abnormalities in control subjects were considered SCI in agreement with most current SCA literature. However, these lesions could also be reported as white matter hyper intensities (WMH), which have been the subject of numerous studies in healthy adults (51, 52). While the term has also been used in some SCA literature (25, 53), the vast majority of studies use the term ‘SCI'.

In this study, we found that SCI definition had a significant effect on the mean χ and R${}_{2}^{*}$ of the segmented SCI. Applying the T1w-threshold increased the magnitude of the χ and R${}_{2}^{*}$ differences relative to normal appearing white matter. These differences suggest that the T1w-based segmentations may be more sensitive to regions of abnormal χ and R${}_{2}^{*}$ relative to NAWM, which may reflect necrosis, compared to the FLAIR based definitions. If the presence of SCI were linked to cognitive impairment in patients with SCA, it may be expected that SCI which persist after applying the more stringent T1w-based threshold would have a greater impact on cognitive performance, given the larger differences relative to NAWM. However, no significant association between the presence of SCI, which met the stringent T1w-based SCI definition, and cognitive performance was found in the previous study in this cohort (15).

MRI signal abnormalities were identified and labeled as SCI based upon two definitions applied in previous studies of sickle cell anemia, considering T2-weighted FLAIR and T1-weighted MP-RAGE contrasts. In the broader ischemic stroke literature, diffusion weighted imaging (DWI) has been used to identify the presence of acute ischemic lesions (54, 55). Acute lesions appear hyperintense on DWI images, and hypointense on maps of apparent diffusion coefficient (ADC). Acute silent cerebral ischaemic events (ASCIE) have been investigated in patients with SCA, and it has been shown that not all ASCIE develop into chronic infarcts (56, 57). The use of FLAIR and T1-weighted image contrasts for SCI identification and segmentation ensured that this study focussed on chronic SCI lesions.

### Investigating SCI χ and R${}_{2}^{*}$ as a function of subject demographics, anatomical location and volume

The greater SCI-NAWM R${}_{2}^{*}$ difference observed in patients with SCA relative to controls (Figure 6) indicates that the myelin content reduction in SCI may be more severe in these patients. This may be secondary to the hemodynamic stress observed in patients with SCA (24, 58). The increased SCI-NAWM R${}_{2}^{*}$ differences in SCI in patients with SCA relative to controls may also reflect the larger of proportion of SCI which persist using the T1w-based definition in patients with SCA. The reduced R${}_{2}^{*}$ of the SCI segmented in patients with SCA relative to controls show that there are larger differences in SCA SCI relative to NAWM. This suggests that more white matter damage/necrosis occurs in SCI in patients with SCA relative to controls, and that lesions in patients with SCA and controls may arise from different mechanisms.

No significant association between SCI–NAWM χ or R${}_{2}^{*}$ difference was observed with age in this cohort. If the mechanisms for SCI were dynamic and lesion composition evolved with time, then we might expect to see such associations. However, their absence suggests that negligible changes in lesion composition occur within the lesion once it has been sustained. It may be more valid to investigate lesion composition as a function of lesion age as opposed to subject age, to determine if changes in lesion composition occur with time. Note that the age of the infarcts in this study is not known and, therefore, the χ of the SCI could not be investigated as a function of lesion age. Future, longitudinal studies would allow examination of the formation of new lesions and evaluation of their evolution with time.

Both SCI and NAWM χ did have a significant negative association with the log-transformed patient age, and R${}_{2}^{*}$ values showed a significant positive association for the FLAIR-based segmentations (Figure 5). In the NAWM, decreasing χ and increasing R${}_{2}^{*}$ with age is consistent with increasing myelin content, as expected in these young subjects with active myelination. The results of the SCI–NAWM comparison showed reduced susceptibility sources in the SCI relative to NAWM. However, the associations of SCI χ and R${}_{2}^{*}$ with age suggest that some myelin content remains in these regions and that the amount of myelin increases as a function of age. If there were no myelin present within the segmented SCI, we would expect no relationship with age. The SCI, and corresponding NAWM, segmented using the T1w-based definition demonstrated no significant associations with age (Supplementary Figure 2), probably due to the low number of lesions persisting after the more stringent definition.

No significant effect of lesion position in the brain (neither brain lobe nor lesion depth in white matter) was observed on mean SCI–NAWM χ or R${}_{2}^{*}$ difference (Figure 7, Supplementary Figures 4, 5).

While significant differences were observed between mean χ in NAWM in the frontal and parietal lobes, no such differences were observed between R${}_{2}^{*}$ values. Similar differences between lobes may be expected for both for the χ and R${}_{2}^{*}$ values, as they are both dependent upon the concentration of susceptibility sources. The discrepancy between R${}_{2}^{*}$ and χ values may have been caused by differences in the χ anisotropy of the regions or residual background fields present only in the χ maps. Emerging QSM techniques such as weak-harmonic QSM can be used to remove residual background fields when calculating χ maps and could help isolate the reason for this discrepancy between the χ and R${}_{2}^{*}$ results (59).

Differences in the mean SCI χ/R${}_{2}^{*}$ as a function of white matter depth may have been expected if differences in lesion composition were present. In this predominantly SCA cohort, a large proportion of the lesions were present in deep and juxtacortical white matter, which differs from WMH in elderly healthy populations where the majority of lesions are found in periventricular regions (60).

In QSM, χ is assumed to be an isotropic property which is independent of the direction of the applied magnetic field. This assumption is known to break down in white matter, where the highly structured axons result in susceptibility anisotropy (61–63). As a result, when comparing lesions in different regions of the brain, changes in χ could be caused by differences in orientation relative to the main magnetic field, in addition to changes in tissue composition.

It may be more informative to examine the white matter tract in which the lesion resides, as opposed to considering the lesion depth within white matter. This could be achieved by incorporating information from diffusion tensor imaging (DTI). Furthermore, spatial variation could be considered by examining the position of the SCI relative to the arterial territories. SCI are most commonly reported in the border zone region between arterial territories (11); however this finding comes mostly from low-resolution studies, and our recent high-resolution study suggests that smaller SCI may be just as common in juxta-cortical regions (64).

### Investigating effect on cognitive impairment as a function of SCI χ and R${}_{2}^{*}$

It has previously been widely reported that the presence of SCI is linked to increased cognitive deficits in patients with SCA (8). However, our previously published data in this cohort found no significant associations between measures of cognitive performance (FSIQ, WMI, and PSI) and conventional SCI lesion metrics (binary lesion presence/absence, number of lesions, and lesion volume) for any of the lesion definitions considered (15). Here, we investigated whether SCI tissue characteristics i.e., χ and R${}_{2}^{*}$ are associated with cognitive impairment and found no associations.

The cognitive difficulties present in patients with SCA have been reported to worsen with age. In the general population, white matter hyperintensities appear to be associated with longitudinal intellectual decline, particularly in those with mild cognitive impairment and stroke (65). Autopsy data are consistent with an effect on myelin secondary to arteriosclerosis and ischemia (66), associated with decline in perceptual speed, with some evidence of amyloid-β accumulation eventually (67). There are however few MRI data on WMH composition with which to compare our SCI data.

### Limitations

A limitation of this study is the differences in resolution between the three MRI images used i.e., FLAIR: 0.65 × 0.65 × 1 mm^3^, T1w MP-RAGE:1 × 1 × 1 mm^3^, ME-GRE:1.15 × 1.15 × 1.15 mm^3^. The SCI segmentations underwent a series of transformations and resampling with nearest neighbor interpolation before mean χ and R${}_{2}^{*}$ values were calculated in the native space of the tilt-corrected multi-echo gradient echo images. These interpolations often resulted in the loss of voxels from the SCI segmentations (e.g., see Figure 1) and introduced registration errors, labeling non-SCI voxels as lesions. Future studies should aim to utilize more consistent fields of view and voxel dimensions across the multiple MRI sequences acquired.

Partial volume effects will be present in this study due to the affine transformations applied to the lesion segmentations and the different resolutions of the three image contrasts. Partial volume effects may have led to the inclusion of normal appearing white matter tissue within the SCI segmentations. This would be expected to reduce SCI-NAWM differences in both χ and R${}_{2}^{*}$.

Partial volume is likely to have a greater effect on larger SCI segmentations. A significant negative correlation was observed between log-transformed lesion volume and mean SCI χ. This correlation may be affected by the inclusion, in larger segmentations, of normal appearing white matter, which is more diamagnetic than regions of infarction according to the SCI-NAWM comparison.

The effect of applying the T1w-threshold significantly reduced the volume of the SCI segmentations. Applying the T1w-based threshold increased the mean χ and reduced the mean R${}_{2}^{*}$, increasing the SCI χ and R${}_{2}^{*}$ differences relative to NAWM. We might expect the lesions that persist after T1w-based thresholding to have more severe composition changes relative to NAWM, which would explain why these lesions also appear hypointense on T1-weighted images. However, the reduced volume is also less likely to contain partial volume effects so these may also contribute to the significant difference observed between lesion χ and R${}_{2}^{*}$ before and after the application of the more stringent lesion definition.

From the SAC and POMS studies 275 SCI lesions were present in subjects with good quality images from the three necessary MRI contrasts. Following interpolation into the native QSM space, 64/275 lesions had ≤ 2 voxels and were, therefore, excluded. Therefore, the results of this study will contain a smaller contribution from lesions consisting of just of few voxels in the original segmentations, as larger lesions were more likely to persist and be included in the final results

Although the ME-GRE sequence acquired in this study had a final echo time of 27 ms, which is shorter than the mean T2^*^ of the SCI measured in this study (61 ms), the sequence had regular sampling of the magnitude signal decay and, therefore, provided accurate SCI R${}_{2}^{*}$ measurements (68).

After quality control and interpolation into the native QSM space, SCI were examined in 6 healthy controls, compared to 32 patients with SCA. The greater number of patients with SCA recruited in the SAC and POMS studies, relative to the number of controls, and the greater incidence rate of SCI within SCA relative to controls in the participants, means that the results of this study are dominated by SCI in SCA. This study found significant R${}_{2}^{*}$ differences between SCI in patients with SCA and controls. To increase confidence in this result, future work should look to compare SCI within a more evenly distributed cohort of SCA and control participants.

Participants imaged in this study were aged 8.5–63.9 years, with the majority of participants aged between 15 and 25 years. There were few data in participants aged between 25 and 50 years old. To fully understand changes in SCI lesion composition as function of age, future studies should increase the number of SCI investigated in participants within the range 25–50 years.

Regions of normal appearing white matter were positioned in the contralateral side, making sure to avoid the locations of identified infarcts. However, diffusion tensor imaging studies have shown that in some white matter regions, white matter integrity is decreased in patients with SCA relative to healthy controls, even in the absence of any focal regions of white matter damage (69). Therefore, regions which appeared as visually normal on the T1-weighted images and were, therefore, classified as normal appearing white matter (NAWM), may have shown changes associated with SCA.

## Conclusion

R${}_{2}^{*}$ and quantitative susceptibility mapping were used to non-invasively investigate the composition of SCI relative to normal appearing white matter in patients with sickle cell anemia and controls. We demonstrated that SCI were significantly less diamagnetic and had lower R${}_{2}^{*}$ relative to NAWM, suggesting lower myelin concentrations and/or increased water content in both patients and controls. The SCI-NAWM R${}_{2}^{*}$ decrease observed within SCI was significantly larger in patients with SCA compared with controls. SCI definition had a significant effect on mean lesion χ and R${}_{2}^{*}$; in SCI which persisted after the more stringent T1w-based SCI definition was applied, mean R${}_{2}^{*}$ was significantly reduced and mean χ significantly increased. Thus, the T1w-based segmentation appears to be more sensitive to changes in SCI χ and R${}_{2}^{*}$ relative to NAWM. We have shown that future SCI studies can use quantitative MRI methods such as R${}_{2}^{*}$ and QSM to enhance our understanding of the pathophysiology and composition of SCI in patients with SCA as well as controls.

## Data availability statement

Full anonymized data will be shared on request from the corresponding author.

## Ethics statement

The studies involving human participants were reviewed and approved by West London NHS (05/Q0408/42, 11/EM/0084, 15/LO/0347), Yorkshire NHS (15/YH/0213) and University College London (14475/001) Ethics Committees. Written informed consent to participate in this study was provided by the participants' legal guardian/next of kin.

## Author contributions

RM: manuscript preparation, QSM/R2^*^ processing, and contralateral NAWM ROI segmentation. HS: data acquisition, SCI segmentation, and manuscript review. JK: data acquisition. DS: manuscript review and SCI detection. FK: manuscript review and data acquisition. KS: manuscript review and QSM/R2^*^ processing. All authors contributed to the article and approved the submitted version.

## Funding

HS was funded by Action Medical Research (GN2509) and JK was funded by Great Ormond Street Children's Charity (V4615). This work was supported by the National Institute for Health Research and Biomedical Research Center at Great Ormond Street Hospital for Children NHS Foundation Trust and the Institute of Child Health (IS-BRC-1215-20012). KS was supported by European Research Council Consolidator Grant DiSCo MRI SFN 770939. The National Institute for Health Research (UK; PB-PG-1112-29099) and NHLBI (R01HL079937) provided funding for patient recruitment. RM was supported by the EPSRC-funded UCL Center for Doctoral Training in Medical Imaging (EP/L016478/1).

## Conflict of interest

FK was grant holder for GN2509, V4615, PB-PG-1112-29099, and R01HL079937 and has received honoraria from Global Blood Therapeutics, Bluebird Bio, Novartis, BIAL, Shire and Johnson and Johnson outside this work and not relevant to it. The remaining authors declare that the research was conducted in the absence of any commercial or financial relationships that could be construed as a potential conflict of interest.

## Publisher's note

All claims expressed in this article are solely those of the authors and do not necessarily represent those of their affiliated organizations, or those of the publisher, the editors and the reviewers. Any product that may be evaluated in this article, or claim that may be made by its manufacturer, is not guaranteed or endorsed by the publisher.
