# Supplementary material for: Quantitative susceptibility mapping (QSM) and R2* of silent cerebral infarcts in sickle cell anemia
Source: Front Neurol. 2022 Oct 20;13:1000889. doi: 10.3389/fneur.2022.1000889 (PMC9632444; doi:10.3389/fneur.2022.1000889)
Supplement: Supplementary file 1 [file Data_Sheet_1.docx]

Supplementary Material

Supplementary Figure 1: **Effect of SCI Definition on SCI-NAWM χ and R_2_^*^.** Comparison of silent cerebral infarct (SCI) and normal appearing white matter (NAWM) χ and R2* in lesions which do **(a)** & **(d)** and do not persist **(b)** & **(e)** after the application of a T1-weighted based threshold. In the lesions which do persist the T1w-based threshold, the Bland Altman analysis is repeated in the lesions after application of the threshold **(c)** & **(f)**. The mean bias (Δ) and limits of agreement are annotated in each figure.


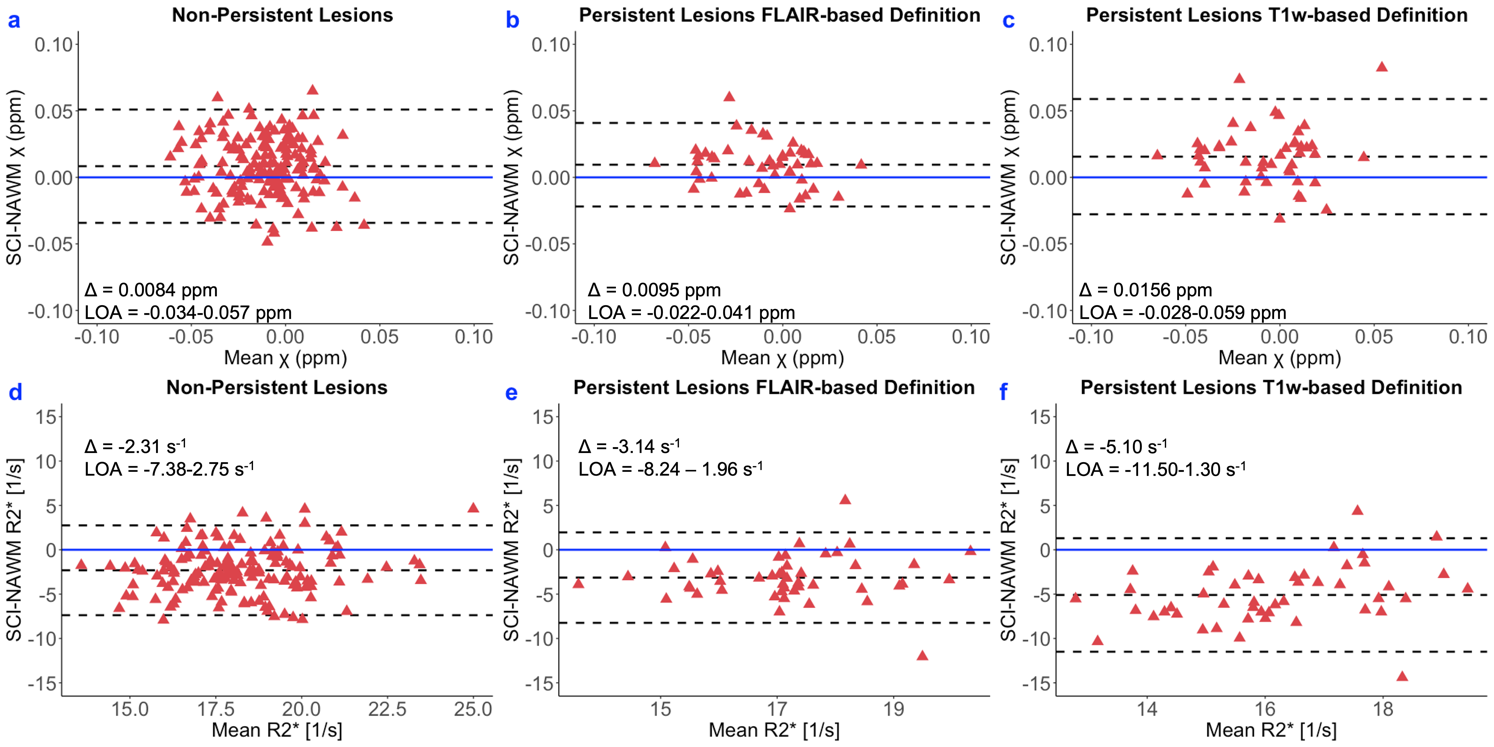


Supplementary Figure 2: **Correlations Between SCI and NAWM χ and R_2_^*^ and Age.**  **(a)** Mean silent cerebral infarct (SCI) susceptibility (χ) **(b)** mean normal appearing white matter (NAWM) χ **(c)** SCI-NAWM χ difference **(d)** Mean SCI R_2_^*^ **(e)** Mean NAWM R2* **(f)** Mean SCI-NAWM R2* difference as a function of age plotted on a logarithmic scale.SCI were segmented based on the T1w-based definition.


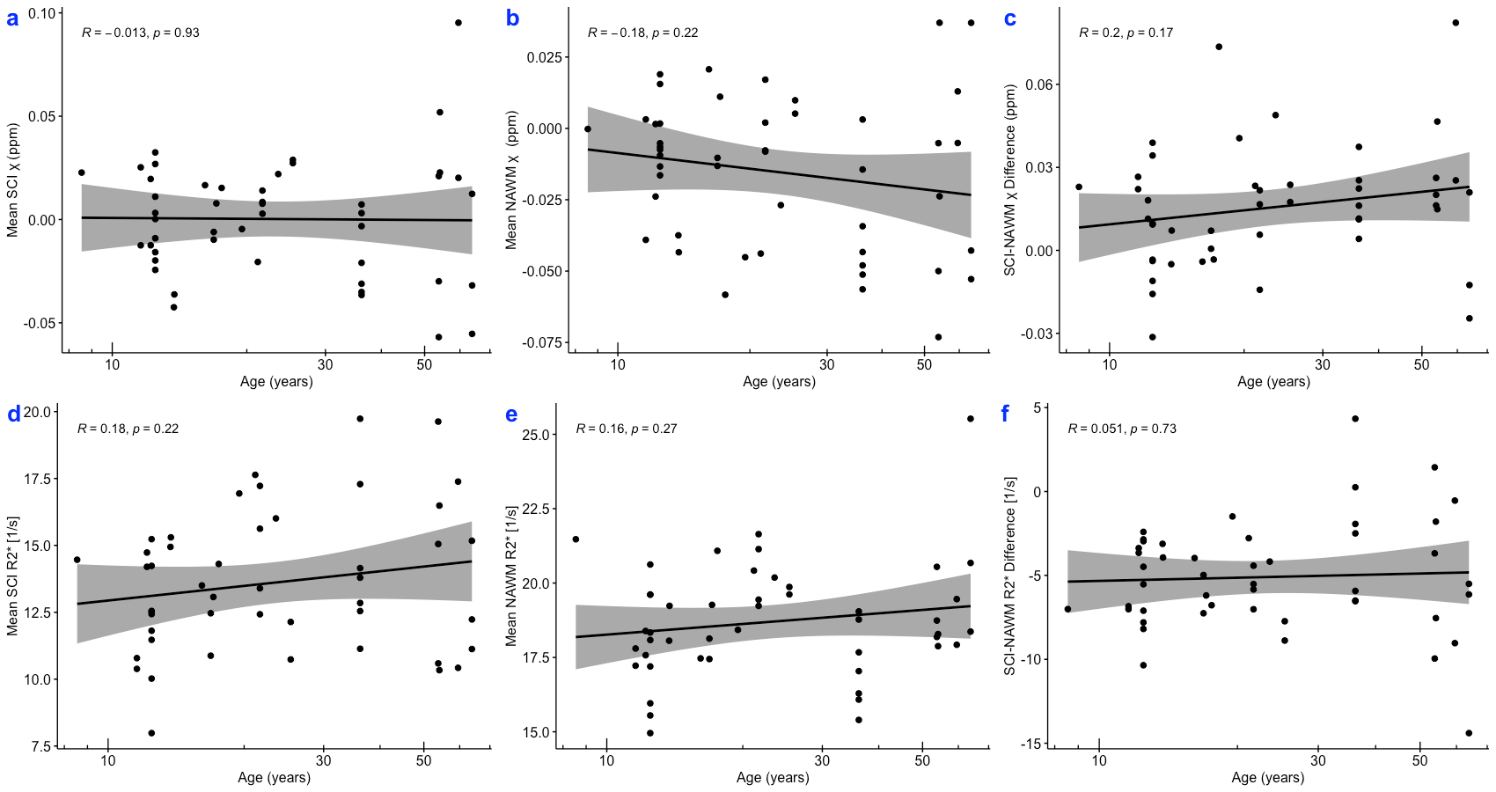


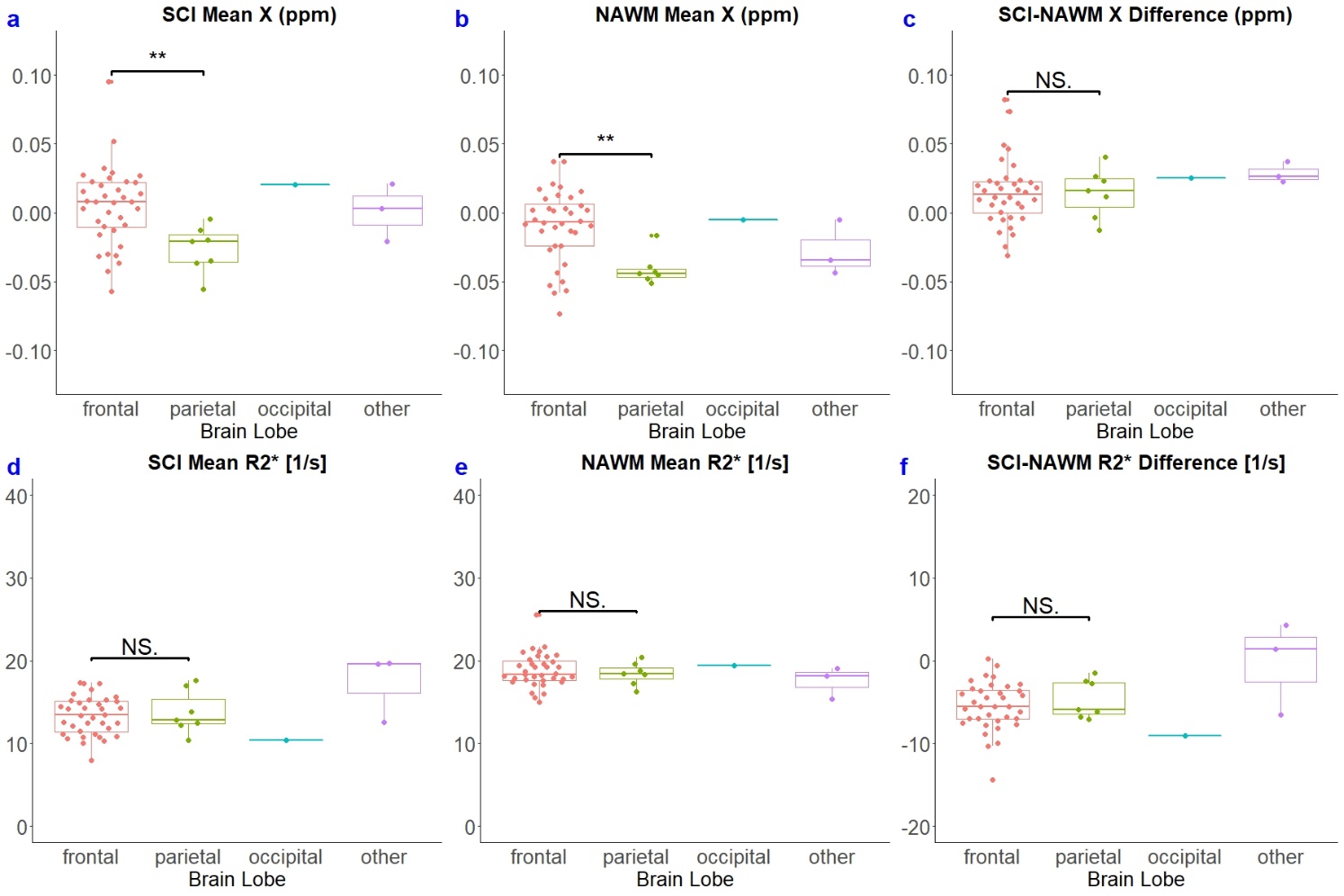
Supplementary Figure 3: **Effect of Anatomical Location on SCI and NAWM χ and R_2_^*^.** Comparison of mean susceptibility (χ) and R_2_^*^ measured in silent cerebral infarcts (SCI) in each of the brain lobes. **(a)** SCI in the frontal lobe were significantly more paramagnetic relative to lesions in the parietal lobe (b) This lobar χ difference was also observed in normal appearing white matter (NAWM). **(c)** This resulted in no significant differences in the SCI–NAWM susceptibility difference between the lobes. **(d) & (e)** No significant differences were observed between lobes in the SCI and NAWM mean R2* comparisons. **(f)** No differences were observed between the R2* SCI-NAWM difference between lesions in the frontal and parietal lobes (f). SCI were segmented based on the T1w-based definition.

Supplementary Figure 4: **Correlations Between SCI and NAWM χ and R_2_^*^ and Lesion Volume.**  **(a) & (d)** Mean silent cerebral infarct (SCI) **(b) & (e)** normal appearing white matter (NAWM) **(c) & (f)** SCI-NAWM differences in susceptibility (χ) and R2* as a function of lesion volume plotted on a logarithmic scale. Mean SCI χ and R2* in regions segmented based upon the more stringent T1w-based definition.


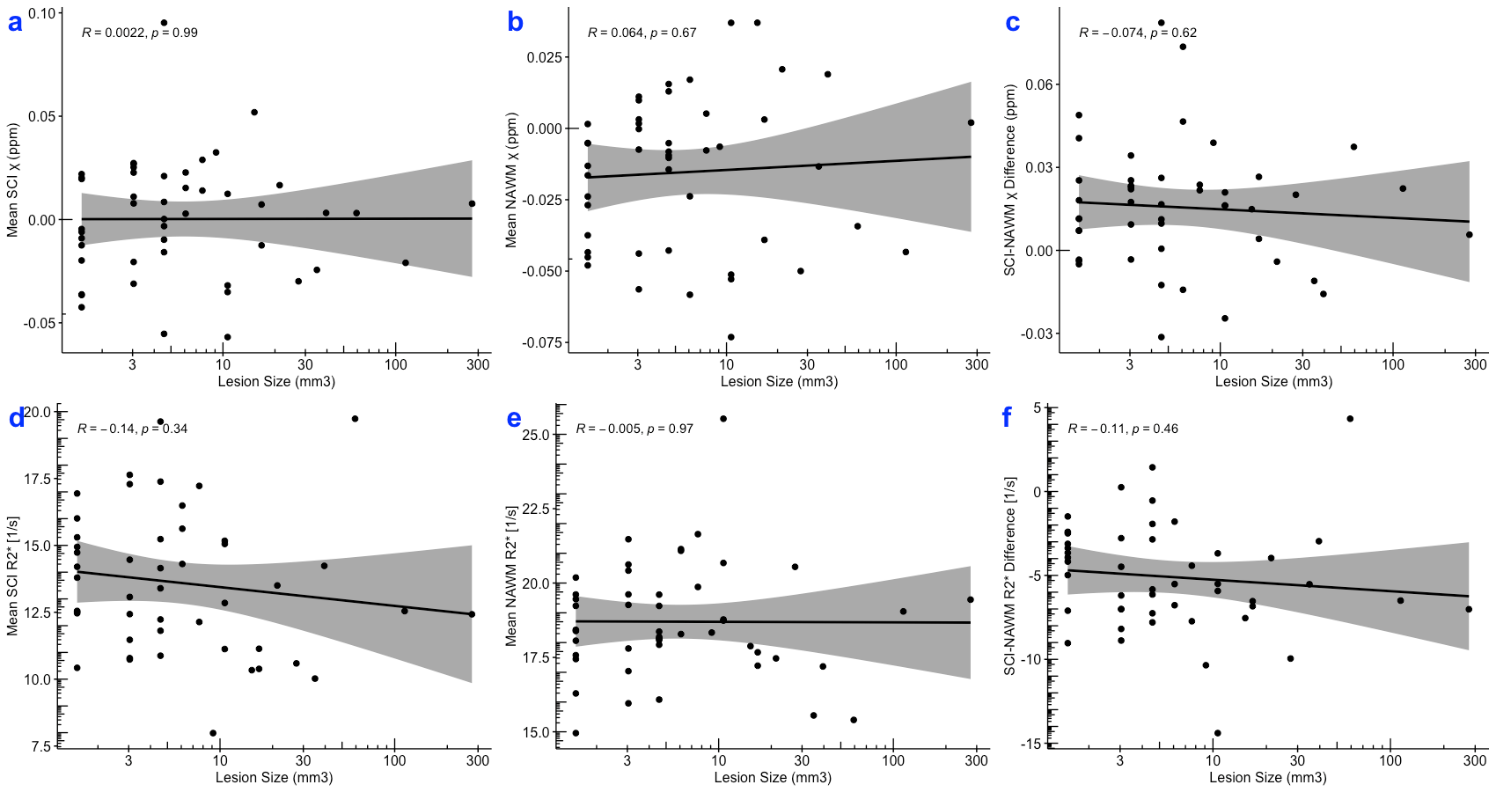


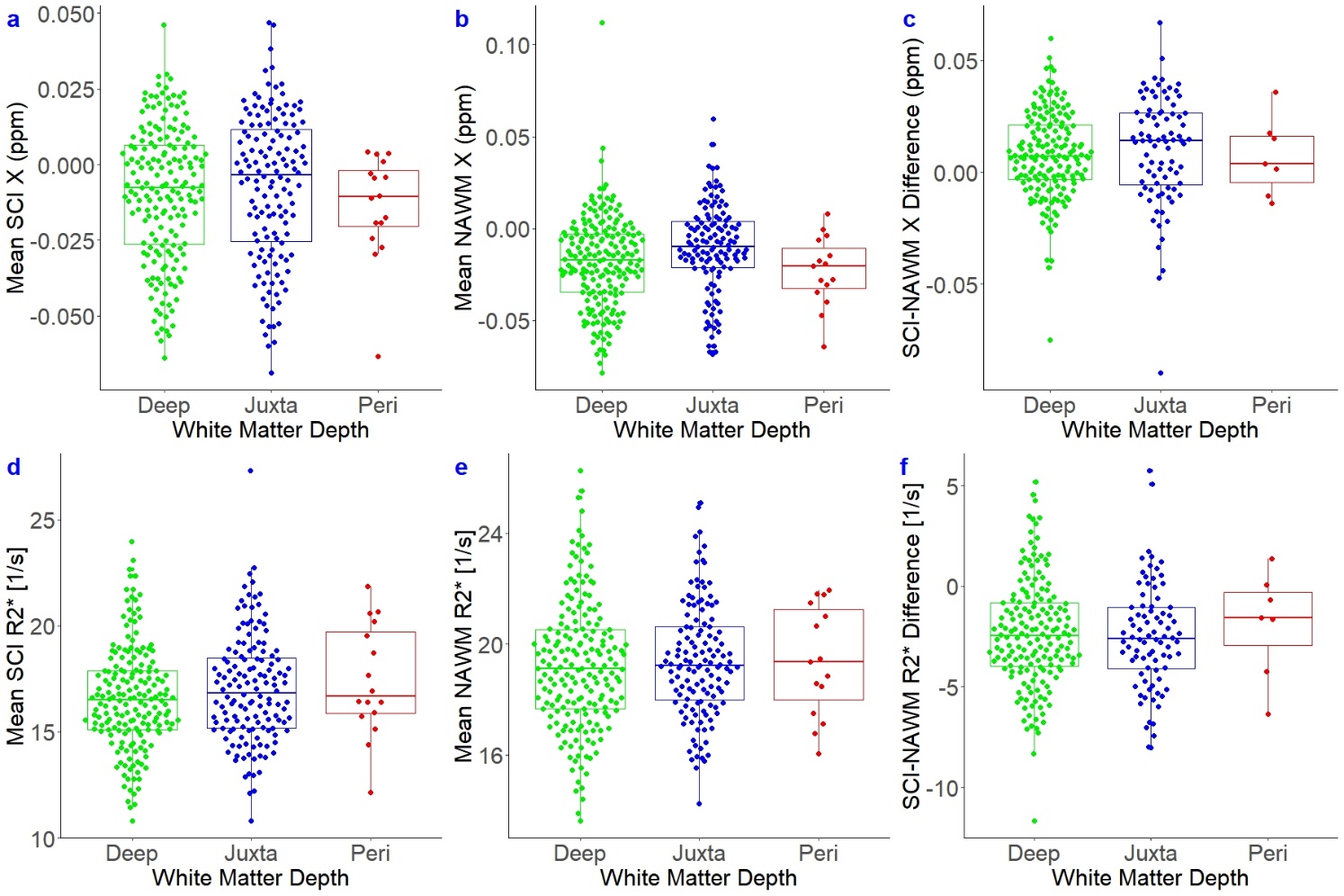
Supplementary Figure 5: **Effect of White Matter Depth on SCI and NAWM χ and R_2_^*^.** **(a)** & **(d)** Mean silent cerebral infarct (SCI) **(b)** & **(e)** normal appearing white matter (NAWM) **(c)** & **(f)** SCI-NAWM differences in susceptibility (χ) and R2* as a function of white matter depth. No significant relationship between the SCI–NAWM χ and R_2_^*^ difference and white matter depth were observed for the juxta-cortical, deep, or periventricular SCI. Mean SCI χ and R2* in regions segmented based upon the FLAIR-based definition.
